# Mechanism and Regulation of Silique Dehiscence, Which Affects Oil Seed Production

**DOI:** 10.3389/fpls.2020.00580

**Published:** 2020-05-20

**Authors:** Yan-Kun Yu, Yu-Long Li, Li-Na Ding, Rehman Sarwar, Feng-Yun Zhao, Xiao-Li Tan

**Affiliations:** Institute of Life Sciences, Jiangsu University, Zhenjiang, China

**Keywords:** silique dehiscence, agronomic traits, phytohormone, signal transduction, agriculture production

## Abstract

Silique dehiscence is an important physiological process during natural growth that enables mature seeds to be released from plants, which then undergo reproduction and ensure the survival of future generations. In agricultural production, the time and degree of silique dehiscence affect the harvest time and processing of crops. Premature silique dehiscence leads to seeds being shed before harvest, resulting in substantial reductions to yields. Conversely, late silique dehiscence is not conducive to harvesting, and grain weight and oil content will be reduced due to the respiratory needs of seeds. In this paper, the mechanisms and regulation of silique dehiscence, and its application in agricultural production is reviewed.

## Introduction

Silique dehiscence is of great significance, as it is the process by which seeds are released from mature siliques. Silique dehiscence is required to expand the growth range of offspring plants, and to provide favorable conditions for prospering species and enriching plant adaptability. Silique dehiscence also plays an important role in plant reproduction and maintains biodiversity among species. However, the specific mechanisms by which plants perform the dehiscence process are not clear.

## Morphology and Dehiscence of *Arabidopsis Thaliana* Siliques

*Arabidopsis thaliana*, a model plant, which has seed-shattering characteristics and was used to study silique dehiscence (Spence et al., 1996). Studies have shown that the development of early flowers and siliques in *A. thaliana* can be divided into 20 stages (Smyth et al., 1990). Early flower development occurs from stage 1 to 12, and silique development occurs from stage 13 to 20. The corresponding characteristics of each stage have also been described (Jeffrey and Hill, 1989; Smyth et al., 1990; Robinson-Beers et al., 1992; Spence, 1992; Bowman, 1994; Sessions, 1997; Ferrandiz et al., 1999).

**FIGURE 1:**
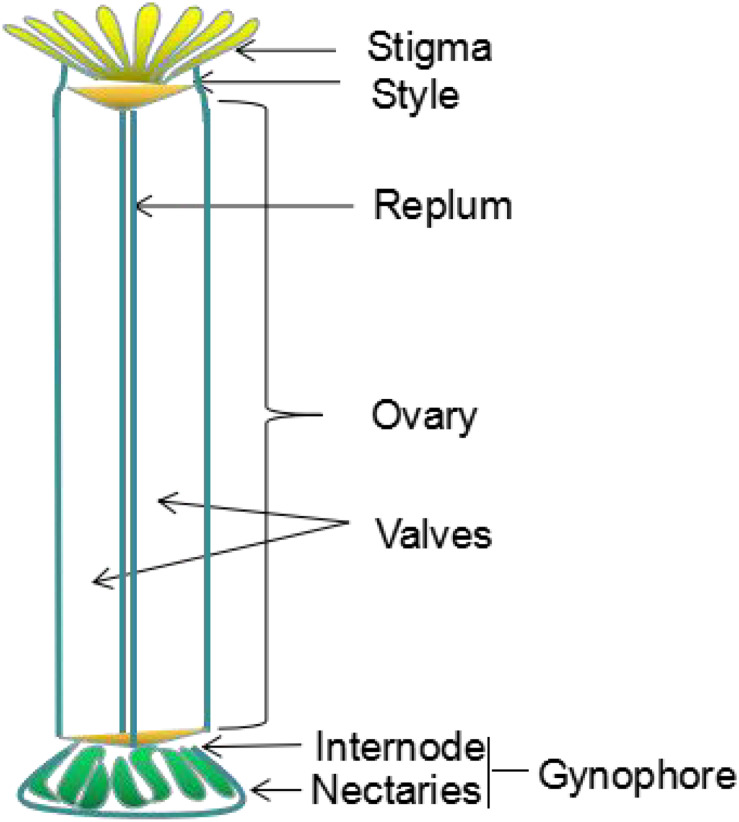
Schematic diagram of gynoecium organization in *Arabidopsis thaliana*, the different parts are indicated. Revised from Ferrandiz et al. (1999).

The *Arabidopsis* silique develops from a gynoecium composed of two fused carpels, which consists of a stigma, style, ovary and gynophore (internode and nectaries) (Figure 1; Ferrandiz et al., 1999). The dried silique is called the silique (Bowman, 1994; Schmid and Rollins, 1994; Sessions, 1999). The silique contains an ovary with two chambers consisting of two linear valves, a replum, and a putative septum between the two valves and valve margins (Bowman et al., 1999). The valve develops from the ovary wall and encloses the seed. It then elongates as seed develops, and separates from the separation layer when the silique is mature; thus resulting in the seeds being released from the dehiscent silique (Smyth et al., 1990).

**FIGURE 2:**
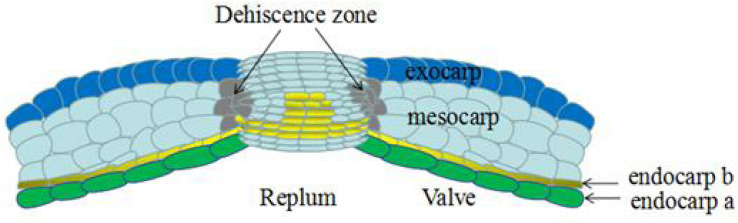
Transversal section of mature silique in *Arabidopsis thaliana*, the different parts are indicated. The silique dehiscence from dehiscence zone, which consists of a lignified layer and a separation layer (Not shown in the figure). Revised from Rajani and Sundaresan (2002).

Previous studies have shown that the dehiscence of the *A. thaliana* siliques is mainly related to the lignification of the endocarp layer and valve margin (Liljegren et al., 2000). The *A. thaliana* silique is composed of two layers of valves, a replum, a septum, and the dehiscence zone (DZ) between the valves and the septum. The silique valve usually contains six cell layers. The outermost layer, called the exocarp, is a single rectangular layer of cells interspersed with immature, unopened stomata, which are required for respiration and transpiration. The following three layers, called the mesocarp, are composed of thin-walled cells containing chloroplasts, and can perform photosynthesis and provide nutrition during seed development. The endocarp consists of two differentiated cell layers: an inner epidermis with large, isodiametric, thin-walled cells (endocarp a), and a subepidermal layer of small and tightly packed cells (endocarp b), which can be elongated longitudinally and lignified (Rajani and Sundaresan, 2002; Figure 2).

Seed spreading is accomplished by silique dehiscence, a process that involves a programmed series of events, during which the valves separate from the replum to release the seed. This process requires the prior development of the DZ at the valve margin, followed by the cells separating in the separation layer. The valve margin consists of a narrow band of four cell layers in entire silique at the valve-replum boundary. During the process of silique extension, the cells of the valve margin extend more slowly than the valve, thus, causing constriction at the valve margin. The DZ is located at the boundary between the valve and replum. The DZ develops from the cells of the valve margin, including both lignified and unlignified cells (Ferrandiz et al., 1999). During the process of silique ripening, thin-walled cells secrete hydrolase to dissolve the middle-lamella and separate the cells from each other (Meakin, 1990; Meakin and Roberts, 1990). Differential shrinkage between the lignified endocarp b cells and the thin-walled non-lignified cells of the valve create mechanical tensions within the silique, which act in a spring-like manner. As the silique matures, a discrete group of cells in the DZ rupture, and the tension in the silique enables the valve to separate from the replum. Whether it is the shedding of fleshy fruit or the dehiscence of dry fruit, the formation of separation zone is needed (Estornell et al., 2013).

In leguminous plants, the varieties of silique indehiscence are the result of interactive selection under long-term domestication and drought conditions, and the silique dehiscence resistance-associated genes selected in different environments may be different (Parker et al., 2020). Some studies have made an anatomical observation on the ventral suture pods of dehiscence-susceptible and dehiscence-resistant varieties of leguminous plants, the results showed that the structures are quite different. It is also found that RFCV (route from the top of fiber cap cells to the connecting point of the two valves), FCC length (fiber cap cell length), VBA (vascular bundle area), VBT (vascular bundle thickness), and LA (bundle cap slope) are related to silique dehiscence (Tu et al., 2019). A recent study identified 163 SNPs (single nucleotide polymorphisms) associated with silique dehiscence, and identified the candidate gene Glyma09g06290, related to silique dehiscence by soybean genomic association analysis (GWAS) and developed molecular markers for silique dehiscence. These findings enrich our understanding of molecular breeding for silique dehiscence (Hu et al., 2019). And in recent study on rice, it has been found that the apetala2-like transcription factor supernumerary bract (SNB) controls rice seed size and shattering, and they found that a SNB allele gene SSH1 is related to seed shttering. In the SSH1 mutant seed shttering decreases and the grain becomes larger, These data indicating that searching for SNB alleles has the potential to increase rice yield (Jiang et al., 2019). The results of this study not only provide a reference for the genetic regulation mechanism of rice shattering, but also provide important target genes for the molecular design and breeding of rice shedding and yield. This method also provides a reference for the study of silique dehiscence in other species. Some researchers also carried out GWAS and linkage analysis on dehiscence-susceptible and dehiscence-resistant in Chinese rapeseed germplasm. It was found that silique dehiscence was controlled by multiple gene loci, and SNP markers significantly related to silique dehiscence were identified, which provide an effective method for improving resistance of silique dehiscence in rapeseed (Liu et al., 2016). Recently, the evolution of silique dehiscence in different species, including *A. thaliana*, legumes, cereals and rice, has been reported. It is found that phenotypic evolution under domestication may be caused by mutations at homologous sites and different types of fruits show phenotypic assimilation at the tissue level (Di Vittori et al., 2019).

In leguminous plants, silique shattering occurs by two modes: one is active silique shattering, which is independent of external forces, but is dependent on the tension between its own cells. The other mechanism is passive silique shattering or enzyme regulated dehiscence; that is, cell wall modification enzymes in the DZ decompose the cells in the separation zone, leading to silique shattering (Luo et al., 2015). Active silique shattering occurs in *A. thaliana*, oilseed rape and other cruciferous siliques. Upon silique maturation in *A. thaliana*, the inner cells of the valve layer become lignified, while the outer thin-walled cells constrict to produce tension, which cause the siliques to dehisce along the separated layer (Rajani and Sundaresan, 2002). In contrast, the oilseed rape silique is composed of two silique valves, which are linked by a group of narrow, non-lignified cells. Those valves are then pulled in different directions under tension when the siliques are mature (Child et al., 2003). Some studies have found that the arrangement of fibers in the valve and degree of lignification also affect the degree of dehiscence of the silique (Řepková and Hofbauer, 2009; Yang et al., 1990). Cruciferae and leguminous plants have multiple similarities in the mechanisms of silique shattering, which necessitate further study.

Oilseed rape siliques are similar to those of *A. thaliana*, and are composed of two layers of valves, a replum, a septum, and the DZ between the valves and septum. Valves usually contain six cell layers (the exocarp, mesocarp, endocarp a, and endocarp b). The septum, silique stalk, and beak are integral, and the seeds are inserted on the septum. There may also be great similarities to *A. thaliana* in the mode and mechanism of dehiscence based on structure similarities. It has been reported that the dehiscence of oilseed rape siliques not only requires the differentiation and separation of specific cells, but also requires a specific water content in the silique. When the water content of the silique becomes less than 80%, the silique begins to dehisce (Zheng et al., 1999).

## The Genetics of Silique Dehiscence

In *A. thaliana* and oilseed rape, silique dehiscence is the result of natural evolution, which facilitates the spread of seeds. Many genes have been identified as being involved in DZ differentiation and related biochemical and physiological changes prior to cell separation during silique dehiscence. Two main methods were used to detect and identify such genes. One method was to identify mutants by classical gene mutagenesis and reverse genetics (Rajani and Sundaresan, 2002), while the other method used reporter genes to identify genes that were specifically expressed in the DZ. Genes expressed specifically in the DZ were identified mainly by isolating mRNA expressed in the valve margins (Coupe et al., 1994; Petersen et al., 1996; Whitelaw et al., 1999). The collection of enhancer or gene trap lines based on the random insertion of reporter genes in the *Arabidopsis* genome provides materials for this study. When inserted near the enhancer or within the gene, neighboring regulatory sequences would regulate the expression of reporter genes (Sundaresan et al., 1995). Silique dehiscence resistance is a recessive trait, study showed that trait has little variation of dehiscence resistance in *Brassica napus*, but a great variation in *Brassica* crops such as *Brassica campestris*, *Brassica juncea*, *Brassica carinata*, and *Brassica nigra* (Morgan et al., 1998). The dehiscence resistance genes were introduced into *B. napus* by interspecific hybridization and synthetic *B. napus*, so as to select the lines with dehiscence resistance. Researchers hybridized the dehiscence resistance line with the dehiscence sensitive lines, and the subsequent genetic analyses revealed that the additional effect of the gene in the dehiscence resistance traits reached a higher level, and the traits related to dehiscence resistance were stably inherited (Morgan et al., 2000).

## Genes Associated With Cell Differentiation in the *Arabidopsis Thaliana* Silique Dehiscence Zone

### Shatterproof1 (SHP1) and Shatterproof2 (SHP2)

*Shatterproof1* and *Shatterproof2* are transcriptional regulators belonging to the MCMl AGAMOUS DEFICIENS SRF box (MADS-box) gene family, which are involved in various aspects of plant development (Rounsley et al., 1995). They regulate the differentiation of the DZ and promote the lignification of cells adjacent to the valve, as well as promote the formation of the valve margin. Phylogenetic and functional studies revealed extensive functional redundancy among members of the MADS-box gene family (Bowman et al., 1991; Kempin et al., 1995; Purugganan, 1997; Riechmann and Meyerowitz, 1997). *SHP1* and *SHP2* are two such genes with high functional redundancy, and share 87% homology at the amino acid sequence level (Ma et al., 1991; Savidge et al., 1995; Flanagan et al., 2010). There is no significant difference between single mutants of *shp1*, *shp2*, and wild-type *A. thaliana*; however, the *shp1shp2* double mutant exhibited an indehiscent phenotype (Liljegren et al., 2000).

Because lignification within the silique may lead to silique dehiscence (Spence et al., 1996), phloroglucinol was used to detect the lignification pattern in *shp1shp2* and wild-type siliques. Such studies found that, in wild-type plants, the valve margin cells adjacent to the DZ were lignified throughout the silique, while lignification was reduced in *shp1shp2* double*-*mutant plants (Liljegren et al., 2000). Scanning electron microscopy and transverse sections showed that, compared with wild-type siliques, *shp1shp2* siliques exhibited less constriction of the valve margins, fewer lignification cells at the valve margin, and the absence of DZs (Liljegren et al., 2000). These results confirmed that *SHP1* or *SHP2* was necessary for dehiscence in *A. thaliana* siliques. *SHP2* expression is repressed by *FUL* in the valves (Ferrándiz et al., 2000b) and is repressed by *APETALA 1* (*AP1*) in the outer whorls of the flower (Kaufmann et al., 2010). A recent study has led to a further understanding of the transcription factor SHP2. Studies have shown that there are redundant cArG box *cis* elements upstream of *SHP2*, which regulate the expression of *SHP2* in the pistil, valve margin and cleavage zone (Sehra and Franks, 2017).

### Fruitfull (FUL)

*Fruitfull* is a member of the extended MADS-box gene family, which is specifically expressed at different stages of plant development. It is involved in the regulation of flower and silique development in *A. thaliana*. During flower development, *FUL* is first expressed in the carpel primordia, and then accumulates in the flower meristem, which can promote flower initiation and development (Alvarez and Smyth, 1997). During the development of siliques, *FUL* promotes the normal development and differentiation of valve cells, and also controls the extension of the silique (Gu et al., 1998; Ferrándiz et al., 2000a). Studies showed that the most obvious difference between the *ful-1* mutant and wild-type plants was that the length of siliques become shorter in the *ful-1* mutant, with crowded seeds inside (Gu et al., 1998). The phenotype of *ful-1* siliqus was due to the lack of lateral valve expansion, differentiation defects of the outer epidermis of the vale, ectopic lignification of the valve mesophyll layers, and a significantly increased number of inner epidermal cells in valves, resulting in seeds becoming crowded within the smaller silique. Additionally, the carpels ceased development soon after pistil fertilization in the *ful-1* mutant. Compared to the wild-type plants, the number of seeds and the dry weight of siliques both decreased the *ful-1* mutant, while the length of siliques was also significantly reduced (Gu et al., 1998).

The epidermis cells of *ful-1* mutant silique valves were irregularly arranged, small with no stomatal precursor cells, and the boundary between the valves and the replum was not distinct. Additionally, after developmental stage 15, cell differentiation and expansion in the replum and valves did not proceed normally (Bowman et al., 1989). The epidermal cells outside the replum failed to form a parallel arrangement, which otherwise occurs in the wild-type plant, but were arranged in a zigzag shape, perpendicular to the main axis of the silique (Gu et al., 1998).

Studies showed that *FUL* is an upstream regulator of *SHP1* and *SHP2*, which affects the formation of the separation layer and lignification of the valve margin (Liljegren et al., 2000). The *shp1shp2* loss-of-function mutants, and *35S:FUL* gain-of-function line showed similar phenotypes (Benfey and Chua, 1990), and both produced indehiscent fruit. Thus, those data suggest that *SHP* and *FUL* genes may interact antagonistically during valve margin development (Ferrándiz et al., 2000b). And some studies have shown that FUL play its role through cArG boxes, and there are cArG box motifs upstream of SHP2, which further illustrates the inhibitory of SHP2 by FUL (Bemer et al., 2017). The regulatory networks of silique dehiscence is continuously updating, from the basic transcription factors necessary for silique dehiscence zone differentiation to the genes related to the development of leaves, flowers and flanking organs, to plant hormones, which play an important role in the differentiation of silique dehiscence zone. And the core regulatory module SHP-FUL which controls the development of dehiscence zone is highly conservative from the model plant *A. thaliana* to closely related crops in the regulation of silique dehiscence (Dong and Wang, 2015). A recent study proved the conservation of SHPS-FUL module in the dehiscence zone differentiation. They selected two local rape varieties namely “*Pakola*” (dehiscence-resistant) and “*PunjabSarsoon3*” (dehiscence-susceptible) to analyze silique morphology, Greater difference was found in silique morphology, which indicated that there was a certain relationship between silique dehiscence and silique morphology. The homologous gene of SHP-FUL was isolated from *Pakola*. Through sequence alignment and expression pattern analysis, it was found that the sequence was highly conserved and there was no significant difference in expression among different varieties (Khan et al., 2019). These studies show that SHP-FUL is highly conservative in regulating the differentiation of dehiscence zone, and also provides a genetic basis for gene editing and breeding varieties suitable for mechanized harvest in the future.

In addition, silique anatomy and microarray analyses were employed to examine the gene expression patterns and related functional analyses during silique dehiscence in *Arabidopsis*. By the end stage of silique dehiscence, a large number of glycosyl hydrolases were enriched in the DZ. Transcription factor enrichment analyses showed that the MADS domain family transcription factors, SEPALLATA3 and AGL15, may be key regulators of silique dehiscence, targeting glycosyl hydrolases related to cell wall degradation (Jiang et al., 2016).

### Alcatraz (ALC)

The *ALC* gene encodes a protein related to the myc/bHLH transcription factors expressed in the valves of siliques, which is the site of cell separation during silique dehiscence. *ALC* is involved in the recognition and development of the separation layer of silique valve margins (Liljegren et al., 2000; Rajani and Sundaresan, 2002). Similar to the *shp1shp2* double mutant, the *alc* mutant plant also showed indehiscent siliques, which could be opened manually by applying external pressure at the valve margins. By stage 19 in wild-type plants, the siliques turned yellow and the valves gradually separated from the replum to release the seeds (Ferrandiz et al., 1999). However, in the *alc* mutant, valve separation from the replum fails to occur (Rajani and Sundaresan, 2002). The patterns of DZ formation at the valve margin, as well as lignification of the internal valve layer in *alc* mutants was examined by scanning electron microscopy, and exhibited no significant difference compared to wild-type plants. Thus, those results indicated that mutation of the *ALC* gene did not disrupt the formation of the DZ, or lignification of the valve layer (Liljegren et al., 2000).

Because there was no visible difference in the exterior of the *alc* mutants, the internal cells of the DZ were examined. A narrow layer of non-lignified cells (NLC) between the lignification cells and the replum cells that exist in wild-type plants were absent in the *alc* mutant. Evaluations of transverse sections of the non-lignified cell layers in mutant siliques revealed that the ectopically lignified cells of the inner valve margin create a lignified bridge between the lignified inner valve cell layers and the lignified replum vasculature, which prevents silique dehiscence. Similarly, loss of the *ALC* gene leads to the non-lignified replum-like cells to occupy the separation site at the outer valve margin, with the ectopic lignified cells toward the inner margin (Gu et al., 1998).

*Alcatraz* is widely expressed throughout the gynoecium before fertilization and is limited to the valve margin after fertilization. In addition to its functional redundancy with other genes during silique dehiscence, *ALC* is also functionally redundant with genes in other regulatory processes (Gu et al., 1998; Rajani and Sundaresan, 2002). Recently, there is a new discovery on *ALC* that the point mutation of *ALC* homologous gene induced by EMS improves the dehiscence resistance of *B. napus*. This work is carried out in oilseed “*EXPRESS*” with silique dehiscence-susceptible (Braatz et al., 2018a). The point mutation of this gene is expected to be widely used in *B. napus*, thus the yield loss of *B. napus* would be greatly reduced.

### Indehiscence (IND)

*Indehiscence* encodes a unique and atypical basic helix-loop-helix (bHLH) protein. It is known that transcription factors with bHLH domain bind to DNA through residues in the basic region. The helix-loop-helix domain promotes the dimerization of proteins with other bHLH factors to form hetero- or homodimers (Murre et al., 1989). Most *Arabidopsis* bHLH proteins contain a key glutamic acid residue (E) in their basic region, which is responsible for DNA binding. Conversely, IND has an alanine residue (A) in the corresponding site (Fisher and Goding, 1992; Buck and Atchley, 2003; Toledo-Ortiz et al., 2003). ALC, another bHLH protein mentioned above, though, the identity of bHLH domain to IND is lower, they both play an important role in silique dehiscence (Liljegren et al., 2004).

*Indehiscence* is necessary for silique dehiscence and is involved in the differentiation of three necessary cell types required for seed dispersal (i.e., the valve, replum and valve margin) (Liljegren et al., 2004; Wu et al., 2006; Van Gelderen et al., 2016). *IND* is essential for the lignification of valve and the development of valve margin cells (Liljegren et al., 2004). The *ind* mutant exhibited indehiscent siliques, similar to the *shp1shp2* mutant, but also exhibited serious defects, such as indistinctive margins at the silique apex (Liljegren et al., 2000, 2004). Evaluation of the silique section showed that the small cells, characteristic of the separation zone and lignified cell layers at the wild-type margin, were not obvious in the siliques of *ind* mutants, predicting markedly decreased constriction of the margin than that of wild-type plant (Liljegren et al., 2004). The lignification of vascular bundles in the replum and valve layer were unaffected in *ind* siliques; however, the lignified cells were not observed throughout the margins of *ind-2* mutant siliques. As margin lignification is only partially affected in *shp* silique and unaffected in *alc* siliques, those data indicated that IND played an important role in regulating the lignification of margin cells (Liljegren et al., 2000, 2004; Rajani and Sundaresan, 2002).

During the development of siliques in wild-type plants, *IND* is expressed at the inner valve layer and throughout a strip at the margin. Those tissues then become lignified later in silique development. The expression of *IND* in the *ful* mutant line extends throughout the complete valve, which indicates that *FUL* is required to restrict *IND* expression to the valve margins. The length of siliques in *ful* mutants is 25% that of wild-type siliques, while the length of silique in *ind/ful* double mutants is restored. Additionally studies showed that *IND* activity was the cause of the loss of e epidermal cell expansion valves (Liljegren et al., 2004). In wild-type siliques, lignification of a single inner valve layer is considered to be conducive to silique dehiscence, but there are four valve layers in *ful* siliques, resulting in ectopic lignification. However, lignification of the inner valve layer was observed in *ind/ful* siliques, which is normal, and further indicates that the activity of IND is also responsible for ectopic valve lignification (Ferrándiz et al., 2000b).

Bayer CropScience has also been committed to the study of silique dehiscence. A published patent applied by Bayer CropScience shows that *IND* allele gene *IND-A1* or *IND-C1* have been obtained in Brassica. The mutations of *IND* allele gene can effectively reduce silique dehisecnce or delay silique dehiscence until after harvest, and can be used for increasing yield. The patent also provides methods to identify molecular markers associated with reduced or delayed silique dehiscence in a population of dehiscence seed plants (Laga et al., 2012). The patent finally was assigned to BASF(Badische Anilin-und-Soda-Fabrik). Later, they claimed to have acquired *Bnind* and *Bnalc* double mutants with increased dehiscence resistance of rapeseed (Laga et al., 2016). However, the research results of silique dehiscence resistance of other well-known breeding companies remain secret. As a result, smaller breeding companies must make greater efforts to integrate dehiscence resistance into their varieties. Silique dehiscence resistance is an important factor to improve and stabilize the yield of rapeseed. Obtaining rape varieties that are easy to be harvested mechanically will undoubtedly promote large-scale production of rape and bring greater economic value. Recent studies have found that several *Bnind* single mutants obtained in *B. napus* by TILLING technology did not show dehiscence resistance, but the double mutants obtained by hybridization showed strong silique dehiscence resistance, these results indicated that the mutation of *Bnind* is recessive. Based on the analysis of *IND* double mutants, it was found that there was a positive correlation between silique length and dehiscence resistance, and the joint area of replum-valve increased, and the cells in the dehiscence zone became smaller and denser, which required more force to dehisce. This study made an important contribution to acquire *B. napus* with strong dehiscence resistance and provided a new direction for rapeseed breeding (Braatz et al., 2018b). Studies showed that *IND* which plays an important role in regulating valve margin differentiation in *A. thaliana* and its homologous gene *HECATE3*, *SHATTERPROOF1/2*, play a role in regulating reproductive tissue development, and they also involved in network regulated by Gibberellin (GA) and auxin. Studies showed that *IND* promotes the development of pollen and anther by regulating GA and auxin, and these hormones also play a role in the development of valve margin, such as IND regulates the development of separation layer by regulating the level of GA through GA degrading enzyme (GA3OX1) (Kay et al., 2013; Dong and Wang, 2015). Some studies have found that the excessive Lignification of fiber cap cells in the ventral suture is the cellular basis and molecular mechanism of indehiscent pods in leguminous plants. In cultivated soybean, it was found that *SHATTERING1-5* (SHAT1-5) and *PDH1* were located in pod dehiscent QTL. In *Phaseolus vulgaris*, *PVIND1*, homologous to *AtIND*, was located near the quantitative trait locus ST of silique dehiscence. But *PVIND1* may not be directly involved in the regulation of silique dehiscence, because study showed it is not linked to the genotype at the ST locus (Gioia et al., 2013; Dong and Wang, 2015; Parker et al., 2020). How it participates in the regulation of silique dehiscence and whether other homologous genes with *AtIND* are involved still need further research to confirm.

### Replumless (RPL)

*Replumless* encodes a transcription factor belonging to the BEL1-Like (BEL1L) family, which is mainly involved in the development of the replum. Previous studies have shown that BEL1L transcription factors regulate ovule development through the negative regulator *AGAMOUS*, a MADS-box gene that is closely related to the *SHP* gene (Modrusan et al., 1994; Western and Haughn, 1999). Similarly, *RPL* may negatively regulates the *SHP* gene to control the development of the replum (Roeder et al., 2003).

In *rpl* mutants, the overall morphology of silique was similar to that of the wild-type, except that the length was half as long as that of the wild-type. The replum cells of *rpl* mutants are also replaced by a narrow file of cells that resemble the cells located in the valve margin. In order to detect whether the narrow file of cells adopted a valve margin identity, examinations of the known expression patterns of the valve margin molecular markers, GT140 (Sundaresan et al., 1995; Ferrándiz et al., 2000b; Liljegren et al., 2000) and SHP2:GUS (Savidge et al., 1995) revealed that those genes were ectopically expressed in the replum region of the *rpl* mutant (Roeder et al., 2003). Those data proved that replum cells developed into valve margin cells. Since the *SHP* genes are ectopically expressed in the replum of the *rpl* mutant, and also control the development of the valve margin, a *rplshp1shp2* triple mutant was constructed to remove the activity of *SHP*, and to detect whether the ectopic expression of *SHP* caused the replum cells to develop into valve margin cells. The results showed that the replum was restored in the triple mutant; thus, indicating that the ectopic expression of *SHP* genes caused the loss of the replum in *rpl* mutants. Another study also suggested that *RPL* is not a direct factor controlling the formation of the replum, but rather, it is necessary to prevent the ectopic expression of *SHPs* in the replum (Roeder et al., 2003).

It is known that there are three main tissues in *A. thaliana* siliques: the valve, the valve margins, and the replum. Previous studies have shown that the formation and development of valve margins is regulated by *SHP1/2, IND*, and *ALC* (Liljegren et al., 2000). Valve development is controlled by the transcription factor, *FUL* (Gu et al., 1998), which negatively regulates *SHP* to prevent valve cells from adopting the identity of valve margin cells (Ferrándiz et al., 2000b). The development of the replum is negatively regulated by *RPL* to prevent the formation of valve margin cells (Roeder et al., 2003). In conclusion, the expression of *SHP1/2, IND*, and *ALC* genes in the valve margin is negatively regulated by *RPL* and *FUL* to control the development of the valve and replum. *SHP1*/2 positively regulate the *IND* and *AL*C genes. *IND* is involved in the identification and development of the lignification layer and the separation layer at the valve margin, while *ALC* is involved in the identification and development of the separation layer at the valve margin (Dinneny et al., 2005; Tang et al., 2007). Besides, some new factors were identified to involve in silique dehiscence. Recently, it has been revealed that *APETALA2* (*AP2*) regulates *SHP* and *IND*, *RPL* to ensure their proper expression level during silique dehiscence (Ripoll et al., 2011). Some studies have shown that *FILAMENTOUS FLOWER* (*FIL*) and *YABBY3* (*YAB3*) and *ASYMMETRIC LEAVES1/2* (*AS1/AS2*) (Sawa et al., 1999; Eshed et al., 2004; Alonso-Cantabrana et al., 2007) related to lateral organ development, and *JAGGED* (*JAG*) (Dinneny et al., 2004; Ohno et al., 2004) promoting the growth of lateral organs jointly promote the expression of *FUL* and *SHP* (Dinneny et al., 2005). Genes generally associated to meristem-related functions act in the replum and control replum width, such as *BREVIPEDICELLUS* (*BP*), *NO TRANSMITTING TRACT* (*NTT*) or *WUSCHEL-RELATED HOMEOBOX 13* (*WOX13*) (Alonso-Cantabrana et al., 2007; Romera et al., 2012; Marsch-Martinez et al., 2014). Study show that boundary genes, like *CUP-SHAPED COTYLEDON* (*CUC*) and *KNOTTED1-LIKE FROM A. THALIANA 2/6* (*KNAT2/6*), are also expressed in the valve margins (Hepworth and Pautot, 2015).

## Downstream Effectors Associated With Silique Dehiscence

Cellulose, hemicellulose, and pectin are the main components of plant cell walls (Keegstra, 2010). During plant cell differentiation, organ shedding and dehiscence, Cellulase, hemicellulase, and pectinase are responsible for the modification and degradation of these components of cell walls, respectively. Although the biochemical reactions of these enzymes are known, little is known about their biological functions during developmental processes.

The downstream of silique dehiscence is ultimately related to cell wall rupture, cell senescence, and apoptosis, especially the degradation of the middle lamella, which is an important process during dehiscence and is also a feature that is shared with other processes, such as abscission or senescence (Roberts et al., 2000; Patterson, 2001). Cell wall rupture proceeds through the action of enzymes such as cellulases, hemicellulases and pectinases. Several such enzymatic activities have been identified in *A. thaliana*.

The first gene identified as being associated with silique dehiscence encodes a polygalacturonase (PG), a pectinase exist in the DZ of *B. napus* silique, which is called *B. napus* endo-polygalacturonase *(RDPG1, SAC66)* (Jenkins et al., 1996; Petersen et al., 1996). Its homologous genes in *A. thaliana* are *Arabidopsis* endo-polygalacturonases (*ADPG1, SAC70*), which were isolated based on sequence similarity (Sander et al., 2001; Jenkins et al., 2010). The expression patterns of the *RDPG1* promoter in *A. thaliana* transgenic plants (Sander et al., 2001) and the *ADPG1* promoter in *Brassica* plants (Jenkins et al., 2010) were detected and analyzed in heterologous systems. In *Brassica* plants, the reporter gene driven by the *ADPG1* promoter was expressed in regions where cell separation occured such as at the anther dehiscence site and the DZ. Similar results were observed in *A. thaliana* (Roberts et al., 2002). A tomato pectinase, *positional sterility-2* (*PS-2)*, is a homologous gene of *ADPG1*, which is required for anther dehiscence (Gorguet et al., 2006, 2009). Thus, those results show that the function of pectinase in abscission and dehiscence is highly conserved. Studies showed that the expression of *ADPG1* in the silique DZ and the seed abscission zone is regulated by the INDEHISCENT and HECATE3 transcription factors, respectively (Ogawa et al., 2009). In apple and tomato, the expression of the gene encoding pectinase in the pedicel abscission zone is controlled by the MADS-box transcription factors, JOINTLESS (J), MACROCALYX (C), and SIMBP21 (Nakano et al., 2015). PG expression is controlled at the transcriptional and post-translational levels. *PGs* expressed in the silique dehiscence and abscission zones contain a cleavable N-terminal domain, which seems to prevent PG from being targeted to the cell wall. When the plant is triggered by development signals, the cleavable N-terminal domain of PG is removed, and the mature proteins are transported extracellularly to play their roles (Degan et al., 2001). A study show that pectin methylesterases may be associated to the degradation of the middle lamella at valve separation, although there is no direct evidence (Jaradat et al., 2014), A recent study showed that *A. thaliana* ADPG1 is involved in the defense system, which changes the content or composition of lignin through ectopic expression. Thus, ADPG1 could trigger the expression of defense response genes and release of elicitors, and activates cell wall remodeling to maintain cell integrity and prevent external invasion (Gallego-Giraldo et al., 2020).

It is well-known that cellulase and hemicellulase are involved in abscission and dehiscence, as well as other cell separation processes in plants. In plants, cellulases have tissue-specific expression patterns for many development processes, such as tissue expansion, silique ripening or organ abscission (Brummell and Harpster, 2001). Cellulase activity increased during *B. napus* silique dehiscence, although the corresponding genes for such activity have not been determined (Meakin and Roberts, 1990). Similar to pectinase genes, the expression of cellulase genes in the pedicel abscission zone of tomato are also regulated by the MADS-box transcription factors, J, MC, and SIMBP21. The expression of the tomato cellulase gene, *CELLULASE4*, is involved in rapid cell expansion in some organs, such as hypocotyls and leaves (Brummell et al., 1997). Similarly, the cellulase gene, *KORRIGAN*, in *A. thaliana* is also associated with rapid cell elongation (Nicol et al., 1998; Lane et al., 2001). Hemicellulose is another main component of plant cell walls (Heredia et al., 1995). Hemicellulase may have similar effects on plant development as cellulase and pectinase. In the *A. thaliana* genome, there are 25 cellulase genes (Urbanowicz et al., 2007) and eight mannanase genes (Yuan et al., 2007). Recent studies have confirmed that these genes play a role in *A. thaliana* silique dehiscence. The cellulase gene, *CELLULASE6* (*CEL6*) and the hemicellulase gene, *MANNANASE7* (*MAN7*), were isolated from *A. thaliana*, and their functions in the development and dehiscence of *A. thaliana* siliques was elucidated. Those genes were expressed in both vegetative and reproductive organs, and their expressions in the silique was partially regulated by the INDEHISCENT and ALCATRAZ transcription factors. They indirectly affect the time of cell differentiation in valves and promote the degradation of the separation layer cells to facilitate silique dehiscence (He et al., 2018). Xyloglucan endotransglycosylase (XET) is another enzyme involved in cell wall loosening (Fry et al., 1992). Studies showed that during the final stage of silique development in *B. napus*, an XET-encoding gene was up-regulated in the DZ, while an enhancer trap line (YJ8) found in *A. thaliana* appeared to be positively regulated by *SHP1/2* (Roberts et al., 2000; Ferrandiz, 2002). In order to complete silique dehiscence, many enzymatic activities are required, as well as others whose association with dehiscence still need to be detailed. Previous studies have showed that several genes involved in lignification and remodeling of the cell wall also have function in silique dehiscence. For example, NAC SECONDARY WALL THICKENING PROMOTING FACTOR 1 (*NST1*) and related *NST3* regulate a suite of genes involved in lignin and cellulose synthesis (Mitsuda and Ohme-Takagi, 2008). In addition, lignin is a characteristic of differentiation in many cell types, including xylem, and the endocarp b layer of valves, in which the spatial pattern of lignin deposition is closely related to the function of the cells (Roppolo and Geldner, 2012; Hofhuis et al., 2016). Previous studies have shown that lignin forms a honeycomb-shaped structure, which provides a mechanical constraint to limit the diffusion of cell wall-degrading enzymes at the site of cell-cell detachment. Lignin molecular brace playing an important role in organ separation (Yuree et al., 2018). The continuous improvement of gene editing techniques such as CRISPR-CAS9 system and the development of molecular markers will accelerate the research process of silique dehiscence, and finally achieve large-scale mechanized harvest of oilseed rape. At the same time, the research results on silique dehiscence of leguminous plants will also provide reference for oilseed rape silique dehiscence (Ogutcen et al., 2018).

## Signal of Silique Dehiscence

Signal transduction during silique dehiscence is essential for the completion of each event in the process of silique dehiscence. So far, the signaling molecules and mechanisms by which intercellular communication occurs during silique dehiscence have not been clarified. However, some candidate factors can be proposed based on sequence similarity and expressions pattern. Two putative membrane-binding proteins have been identified as participants in silique dehiscence, including *YJ80* and *YJ115. YJ80* is inserted near a gene encoding a mammalian anchor protein-like protein and is expressed at the valve margins and abscission zones of seeds (Ferrandiz, 2002). *YJ115* is inserted into the upstream of a gene unknown function that contains a putative transmembrane domain and is expressed in the abaxial replum and the valve margins (Ferrandiz, 2002). It has been reported that *DEFENSE*, *NO DEATH1* (DND1), which is expressed in the DZ of *Arabidopsis* siliques, encodes cyclic nucleotide-gated ion channel (CNGC) (Köhler et al., 2001). The CNGC is a membrane protein capable of transducing K^+^ and Ca^2+^ ions, and has the same function as other animal CNGCs involved in signal transduction (Leng et al., 1999). One study has also shown that the MADS domain family transcription factors, *SEPALLATA3* and *AG15*, may be involved in *A. thaliana* silique dehiscence by regulating glycosyl hydrolases (Jiang et al., 2016).

Dehiscence and abscission are closely related physiological processes, They all involve the dissolution of intercellular junctions and the degradation of cell walls leading to cell separation (Lewis et al., 2006). And polygalacturonase is needed to loosen the abscission or dehiscence zone cells before cell separation (Ogawa et al., 2009). However, dehiscence and abscission are controlled by different genes in *A. thaliana.* Studies have shown that mutants with flower shedding defects have normal silique dehiscence, on the contrary, there are also mutants with silique indehiscence, whose floral organs shedding normally (Liljegren et al., 2000; Butenko et al., 2003; Patharkar and Walker, 2018). It is reported that a leucine-rich repeat receptor-like protein kinase (LRR-RLK), HAESA, is involved in the regulation of floral organ abscission in *A. thaliana* (Jinn et al., 2000). Some members of LRR-RKs are involved in different developmental processes, but it is not clear whether LRR-RLK is involved in the regulation of silique dehiscence. One study showed that SAC29 is an mRNA specifically up-regulated in the DZ during late silique development in *B. napus*. The protein encoded by *SAC29* is homologous to the receiver domain of response regulator proteins, and may be involved in responding to different stimuli, such as ethylene and cytokinin signals (D’agostino and Kieber, 1999; Whitelaw et al., 1999).

The antagonistic effects of the plant hormones, ethylene and auxin, are known to be involved in abscission (González-Carranza et al., 1998). Ethylene promotes abscission, while auxin delays it. If the delay of silique dehiscence is due to reductions in ethylene, the application of exogenous ethylene can restore the normal timing of cell separation (Child et al., 1998). Increases in cellulase activity in the DZ are due to decreases in auxin concentrations. The application of exogenous auxin analogs delayed DZ cell separation by inhibiting cellulase activity and preventing RDPG1 pectinase secretion into the cell wall (Chauvaux et al., 1997; Degan et al., 2001). Some studies have shown that maintaining auxin minimum in valve margin is necessary for the formation of the separation layer, and this needs to be coordinated by *IND*. *IND* can target several genes related to auxin transport in the valve margin, like *PINOID (PID)* and *WAG2*, which control the distribution of the auxin efflux carrier *PIN-FORMED3 (PIN3)* (Sorefan et al., 2009; Dong and Wang, 2015; Ballester and Ferrandiz, 2017). Studies have shown that cytokinins not only play an early proliferation-inducing role at the medial tissues of the developing gynoecia, but also play a late role in fruit patterning and morphogenesis at the valve margin of developing fruits. The application of exogenous synthetic cytokinins in *shp1*, *shp2*, and *ind* mutants can restore the formation of valve margin, which further show the cytokinins involved in the regulation of silique dehiscence (Marsch-Martinez et al., 2012; Zuniga-Mayo et al., 2014). GA can regulate the differentiation of *Arabidopsis* fruit morphology and silique dehiscence zone, which is also regulated by *IND*. *IND* promotes GA accumulation by regulating gibberellin biosynthesis enzyme GA3OX1, while GA can promote the degradation of DELLA protein bound to ALC, thus releasing ALC to play its role in regulate differentiation of separation layer (Arnaud et al., 2010; Ballester and Ferrandiz, 2017). Abscisic acid may act as a coordination signal (Young and Gallie, 2000). Transcriptome data of *Arabidopsis* siliques development analysis showed that abscisic acid was enriched during silique dehiscence in *A. thaliana*, which confirmed that abscisic acid may play a role in silique dehiscence (Wagstaff et al., 2009). Jasmonic acid (JA) is involved in anther dehiscence. Mutants of JA showed altered timings of anther opening (Sanders et al., 2000; Ishiguro et al., 2001), but there have been no reports on the function of JA in silique dehiscence. However, its positive regulation on flower organ abscission has been proved (Kim et al., 2013). Some studies have shown that salicylic acid (SA) is involved in the process of floral organ abscission, whether it is involved in the silique dehiscence still needs further exploration (Cai and Lashbrook, 2008; Patharkar and Walker, 2015). Recently, studies have shown that auxin controlled by IND specifies the separation layer in *Arabidopsis* siliques (Van Gelderen et al., 2016). In addition, interactions between IND and SPATULA (SPT) promote the development of the separation layer by regulating auxin formation (Girin et al., 2011).

In recent years, the application of plant biostimulants to affect development has achieved preliminary results, but its specific mechanism is not clear (Du Jardin, 2015). Recently, it has been reported that Sealicate, a biological stimulant from the seaweed Ascophyllum nodosum, has effects on the fruit development and seed dispersal of *A. thaliana* and rapeseed. Sealicit was developed utilizing a targeted plant signal induction (PSI) approach to formulation development. Sealicit affects the expression of *IND*, the main regulator of silique dehiscence, and destroys the auxin minimum. These two factors would affect the formation of dehisecnce zone. This provide new direction for the breeding varieties with dehiscence-resistant (Łangowski et al., 2019).

Transcriptome data of *A. thaliana* and tomato fruit development have been analyzed and compared in order to find more similar regulator (Gomez et al., 2014). Preliminary results have been achieved in the application of synthetic plant hormone analogs and related chemical reagents in fruits, such as synthetic auxins and ethylene blockers, which partially block abscission, are sprayed on Citrus and apple trees about a month before harvest (Anthony, 1999; Yuan and Carbaugh, 2007). Plant hormone analogs and related chemical reagents were sprayed before harvest in *B. napus*, the effects of silique dehiscence were observed. If strains which were beneficial to mechanized harvest without affecting their quality were screened, it would show that these chemical sprays have a certain potential in silique dehiscence. With the development of biotechnology and the ability of high-throughput data analysis, the study on silique dehiscence may bring great commercial value.

**FIGURE 3:**
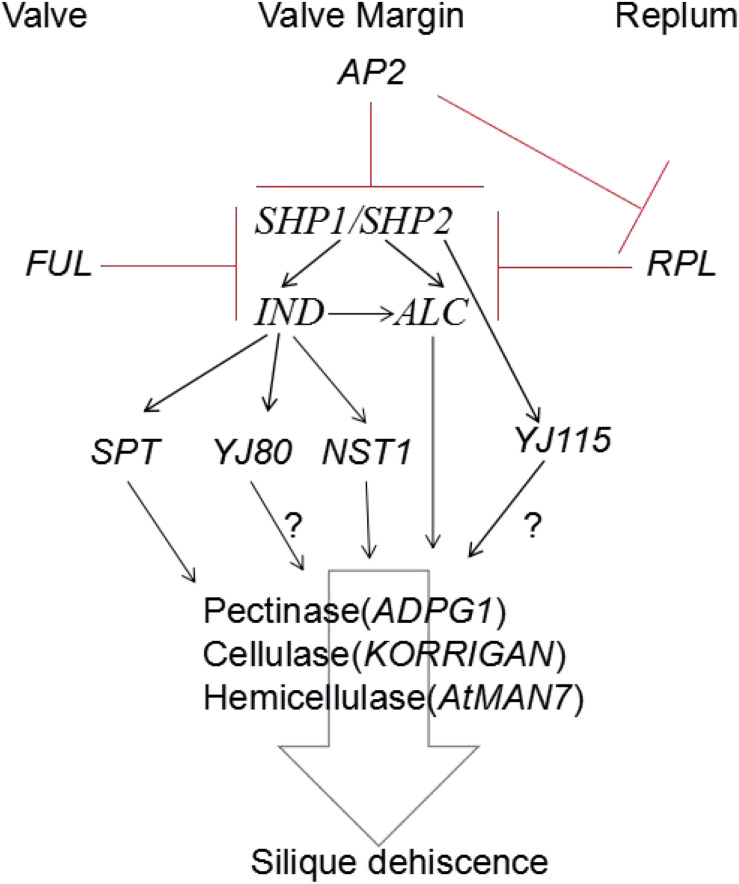
Model for regulation of silique dehiscence in *Arabidopsis thaliana*, The major transcription factors and their regulatory interactions, as well as degradation enzyme of cell wall are included. Hormone coordinate signal transduction in silique dehiscence which not shown in figure. Negative regulation represented by red lines, Positive regulation represented by black arrow. Question marker means hypothetical relationships not well supported by experimental data.

In conclusion, hormones play a role in both DZ differentiation and the coordination of physiological events that lead to cell separation. Few studies have directly addressed these questions so far; however, they can be studied by collecting different *A. thaliana* mutants that have altered hormone synthetic pathways and hormone responses. Based on the findings of previous studies, the mechanism and regulation of silique dehiscence involves many factors that interact in a complex network (Ferrandiz, 2002; Dinneny et al., 2005; Ballester and Ferrandiz, 2017; Di Vittori et al., 2019) (Figure 3).

## Advances From Studies of Silique Dehiscence in *Brassica Napus* Agriculture Production

*Brassica napus* is one of the five major crops, that belongs to Brassicaceae, and can be divided into three categories, including *B. campestris L*., *B. Juncea*, and *B. napus L*., among which *B. napus* has the highest grain yield. In China, *B. napus L*. is mainly planted in the middle and lower reaches of the Yangtze River, and is one of the most widely planted oilseed rape in China, accounting for about 20% of the world’s yield. With the development of the social economy, the demand for, and quality requirements of, oilseed rape are becoming increasingly higher. According to the United States Department of Agriculture (USDA), global edible oil production was 204 million tonnes and consumption was 200 million tonnes in 2018. China produced 26.86 million tonnes of edible oil, consumed 37.67 million tonnes, and imported 8.087 million tonnes in 2018; an increase of 8.9% from 2017. As a major vegetable oil consuming country, a large amount of oil consumption depends on imports, and its production and development are directly related to whether it can meet the edible oil needs of individuals. Taking various measures to continuously increase yield and improve production efficiency is an important goal for oilseed rape research. Mechanized production is an inevitable direction for the development of agricultural production technology. In order to improve the production efficiency of oilseed rape cultivation, improvements in the level of mechanized production are necessary. However, compared with other crops, oilseed rape silique is easy to dehiscence, so it is difficult to realize mechanized harvest.

Researchers have performed long-term explorations of the resistance of oilseed rape silique dehiscence, and developed different methods to detect the character of silique dehiscence. The earliest of such methods was the European Scholars’ Field Investigation Method. The investigation items included the number of seeds lost in the container, the ratio of direct harvest to advance harvest, the number of seedlings germinated by scattered seeds in the field after harvest, the number of seeds scattered in the field, and the number of broken siliques (Kadkol et al., 1984). Although this method is simple to perform, it is time consuming. Moreover, such manual methods are greatly influenced by the investigator’s subjectivity, and are also affected by the environment. Thus, the error in the method is large and the reliability is low. Anatomical methods were used to observe the vascular bundles of oilseed rape (Morgan et al., 1998; Child et al., 2003). It was found that there were large differences in the distribution range, size, quantity and position of vascular bundles in different rape lines. This method overcomes the low reliability of the field investigation method, and identifies the physiologica differences of silique dehiscence traits among different species, but it can only identify obvious differences, and those species with few differences cannot be accurately judged. Additionally, this method is only qualitative, and quantitative. In recent years, a mechanical instrument identification method for simulating the natural dehiscence of siliques in the field has been developed rapidly (Wen et al., 2009).

Researchers proposed a random impact test to detect the resistance of silique dehiscence, the shaking time required to open 50% of dried siliques is tested, but the resistant ability of single silique cannot be detected (Morgan et al., 1998). On this basis, researchers using a shaking table replace the original random collider, adopting multiple collisions, and using the silique dehiscence resistant index instead of the original percentage of broken siliques as an index (Wen et al., 2008). The stability and repeatability of the results were good. In addition, studies reported a new random impact test, in which siliques were baked at 80°C for 30 min to remove the moisture from siliques, which overcame the problem of low resistance to dehiscence in some oilseed rape species (Peng et al., 2012).

Some researchers propose that the dehiscence resistance could be directly evaluated by measuring the tensile force needed for mechanically break siliques (Kadkol et al., 1984; Tys, 1985; Tan et al., 2006). The maximum tensile force at the moment of silique dehiscence represents the dehiscence resistance of the siliques. Other investigators suggested the suspended fracturing method, in which siliques were put on a cantilever beam supporting frame, on which a load force was exerted, and peak force on behalf the resistance of silique dehiscence. The method is simple and rapid, but the reliability and influencing factors must be further explored (Li et al., 2012). Another method of detecting the resistance of oilseed rape to silique dehiscence was designed using a variable speed anti-cracking tester suitable for a single silique on the basis of a single plant thresher. The input current frequency of the silique dehiscence moment was used as an index to measure the resistance of silique dehiscence. When an input current frequency above 60 Hz was applied, the varieties remaining unruptured were regarded as dehiscence-resistant varieties. However, the relationship between this method and dehiscence resistance remains uncertain, and must be verified by further experimentation (Squires et al., 2003). The above-described methods for the identification of oilseed rape silique dehiscence resistance have their own advantages and disadvantages, and further research is necessary to put forward a stable, reliable, time-saving and labor-saving determination method.

The resistance of silique dehiscence is different among different oilseed rape varieties. Some studies reported that the dehiscence of oilseed rape siliques was dependent on the thickness of the pericarp and the degree of development of mechanical tissues in the pericarp (Liu, 1987). The resistance to dehiscence of *B. napus* siliques was the worst, while that of *B. juncea* was second, and *B. campestris* was the highest. Some studies showed that the resistance of siliques was dependent on the length of the siliques, and the content of water and cellulose (Zheng et al., 1999). Studies showed that the dehiscence resistance index of oilseed rape was positively correlated with silique length, pericarp weight, thousand grain weight and seed diameter, and negatively correlated with silique density and the seed number per silique (Cui et al., 2013). A recent study is related to the relationship between replum and silique dehiscence, they study silique morphological structure of rapeseed lines (including dehiscence-susceptible and dehiscence-resistant), it is found that a thick replum structure could produce high silique dehiscence resistance. The replum-valve joint area offers a good method to screen high resistance materials beneficial for breeding (Hu et al., 2015).

The dehiscence resistance of oilseed rape siliques is affected by multiple factors. The most economical and effective way to reduce grain abscission in oilseed rape is to select oilseed rape varieties exhibiting both dehiscence resistance and high yield. Such selection can not only achieve the overall mechanization of oilseed rape harvesting, but also improve the yield of such plants.

## Summary and Prospect of Silique Dehiscence

Silique dehiscence is the result of the joint action of a variety of cell activities, during which a set of physiological and biochemical regulators are involved. First, the early regulators of cell differentiation must play a role in regulating cell specification. Second, after different cell types are determined, disparate enzyme activities must proceed to complete the respective processes, such as changes in cell wall composition, lignification, and the disintegration of the middle-lamella in the separation layer. The differentiation processes and downstream enzymatic activity must be strictly controlled. The signaling mechanism plays a key role in ensuring the coordinated operation of dehiscence-related events.

Controlling silique dehiscence is of great significance in agriculture. For oilseed rape and soybean, it is particularly important to control silique dehiscence and optimize yield. The identification of genes involved in silique dehiscence and the signal regulation mechanism remains a vital problem, for which substantial research still needs to be done. With the completion of the *Brassica* genome sequence, and the ongoing comprehensive analyses of expression patterns and metabolic pathways being performed, useful information may soon be available to provide a better understanding of dehiscence. At present, we are studying the genes related to silique dehiscence in *B. napus L.* with the aim of detailing the molecular mechanisms involved in silique dehiscence.

## Author Contributions

Y-KY was involved in the review writing. X-LT supervised the review. Y-LL, RS, L-ND, and F-YZ was involved in the manuscript refinement. All authors read and approved the manuscript.

## Conflict of Interest

The authors declare that the research was conducted in the absence of any commercial or financial relationships that could be construed as a potential conflict of interest.
